# Phytochemical Index and the Risk of Gastritis/Gastric Ulcer among Korean Adults: A Prospective Cohort Study

**DOI:** 10.3390/nu16152514

**Published:** 2024-08-01

**Authors:** Yeeun Park, Kyong Park

**Affiliations:** Department of Food and Nutrition, Yeungnam University, Gyeongsan 38541, Republic of Korea; yeeun_park@yu.ac.kr

**Keywords:** phytochemicals, gastritis, gastric ulcer, Korea, cohort study

## Abstract

Phytochemicals found in fruits, vegetables, and plant-based foods have potential protective effects against various diseases, including gastric disorders. This study aimed to analyze the longitudinal association between phytochemical intake and the risk of gastritis/gastric ulcer in Korean adults. This was a prospective cohort study, a community-based cohort conducted as part of the Korean Genome and Epidemiology Study, examining the association between phytochemical intake and the risk of gastritis/gastric ulcer in Korean adults. Dietary information was collected using a validated semi-quantitative food frequency questionnaire, and the phytochemical index (PI) was calculated. The study included 7377 Korean men and women aged 40–69 years without gastritis/gastric ulcer at baseline of the Korea Association Resource study in Korea. The incidence of gastritis/gastric ulcer was determined using a survey questionnaire administered by trained staff. Multivariate Cox proportional hazards regression was used to calculate the hazard ratio and 95% confidence interval to determine the association between PI and risk of gastritis/gastric ulcer. During the median follow-up period of 9.50 years, 729 cases were reported. The fully adjusted model showed a significantly lower risk of gastritis/gastric ulcer in the highest PI quartile compared to the lowest (hazard ratio: 0.78, 95% confidence interval: 0.61–0.98), and this association was linear (*p* for trend = 0.01). This research indicates that incorporating foods abundant in phytochemicals into one’s diet could be associated with a reduced risk of developing gastritis/gastric ulcers. These findings underscore the importance of further investigating the role of phytochemical-rich diets in gastrointestinal health, as demonstrated in this study.

## 1. Introduction

Chronic gastritis is a common upper gastrointestinal disorder characterized by prolonged inflammation that can lead to mucosal atrophy and intestinal metaplasia [1,2,3]. The number of patients treated for gastritis in Korea has increased over the years [4], and gastritis and duodenitis were ranked eighth among the most frequent diseases based on outpatient records of a large number of patients [5]. The rate of gastric diseases in East Asia is higher than that in the West, and the age-standardized incidence of gastric cancer is highest in East Asia [6,7,8].

Peptic ulcers, including gastric ulcer, are severe health conditions with serious complications [3,9,10]. The number of patients with gastric ulcers increased considerably after the age of 40 years, with the majority of gastric ulcer patients being in their 50s and 60s [10]. Gastric disorders are one of the major risk factors for gastric cancer, which remains a significant cancer worldwide [7,11,12].

Meanwhile, gastrointestinal diseases, which have not been previously discussed in relation to dietary factors, are also influenced by dietary habits [3,13,14,15,16]. High-salt diet, strong spicy foods, and a pro-inflammatory diet have been linked to an increased risk of gastric disease [11,14,15,17]. A cross-sectional study in China found that several gastrointestinal symptoms in patients with chronic gastritis were strongly associated with a preference for salty or sweet foods and irregular mealtimes [13]. Another prospective study conducted in Korea found that higher energy-adjusted dietary inflammatory index scores, which are associated with a pro-inflammatory diet, were linked to a higher risk of gastric diseases, including gastritis, gastric ulcer, and duodenal ulcer [15]. Studies have also shown that the consumption of fresh vegetables and fruits is associated with a reduced risk of gastric cancer [16,18]. A meta-analysis explored the association between multiple dietary factors and gastric cancer and found that the intake of white vegetables and total fruits was inversely associated with the risk of gastric cancer [16]. These findings suggest that a healthy diet consisting of fresh fruits and vegetables may prevent the development of gastric diseases.

Plant foods rich in phytochemicals offer multiple health advantages, such as antioxidant, anti-inflammatory, and anticancer properties [19,20,21]. Additionally, research has shown the potential protective effects of phytochemicals such as polyphenols and flavonoids against gastrointestinal disorders [22,23,24,25]. However, the association between phytochemical intake and the risk of gastritis/gastric ulcer has not been extensively studied in Asian populations. It is important to address this gap given that the prevalence of gastric diseases is high in East Asia, including Korea [6,7,8]. Recently, the phytochemical index (PI), which reflects the overall phytochemical intake based on the percent of energy derived from phytochemical-rich foods, has emerged as a useful tool for assessing the association between phytochemical-rich foods and various chronic diseases in large community-based epidemiological studies [26,27,28,29,30]. The use of PI has enabled researchers to comprehensively investigate the potential protective effects of phytochemical-rich foods against gastric diseases.

The aim of this study was to prospectively analyze the association between PI and the risk of gastritis/gastric ulcer using data from the Korea Association Resource (KARE) as part of the Korean Genome and Epidemiology Study (KoGES) in Korea. It was hypothesized that a higher PI level would be associated with a lower risk of gastritis/gastric ulcer.

## 2. Materials and Methods

### 2.1. Data Source and Participants

KoGES is a large-scale prospective cohort study conducted by the Korea National Institutes of Health of the Korea Disease Control and Prevention Agency [31]. The present study used data from the KoGES_ KARE, which recruited 10,030 participants aged 40–69 years, between 2001 and 2002 from the general population living in Ansan (city) and Anseong (rural) in Korea; the study prospectively collected data related to diet, lifestyle, disease history, and anthropometric measurements through a baseline survey and in-person follow-ups conducted every two years. This study is a secondary analysis using data from the baseline survey to the fifth follow-up. A detailed description of the KoGES data has been provided previously [31].

A total of 7377 participants were retained for the final analysis following the exclusion of those diagnosed with gastritis/gastric ulcer at baseline (*n* = 2105), those with daily energy intakes below 500 kcal or above 5000 kcal (*n* = 366) [32], and those diagnosed with various tumors at baseline (*n* = 182). Written informed consent was secured from all participants prior to the study, with the data collection and analysis receiving approval from the Institutional Review Board (IRB) of the Korea Disease Control and Prevention Agency and the Ethics Committee of Yeungnam University (IRB number: 202112011-UE003, approved on 18 December 2021).

### 2.2. Demographic and Lifestyle Information

Demographic characteristics and lifestyle information of the participants were collected using a survey questionnaire [31,33]. Information regarding the average monthly household income was collected according to the following categories: <500,000 Korean Republic won (KRW) (<approximately 400 United States Dollar; USD), KRW 500,000–999,999 (approximately USD 400–799), KRW 1,000,000–1,499,999 (approximately USD 800–1199), KRW 1.5–1,999,999 (approximately USD 1200–1599), KRW 2–2,999,999 (approximately USD 1600–2399), KRW 3–3,999,999 (approximately USD 2400–3199), KRW 4–5,999,999 (approximately USD 3200–4799), and KRW ≥ 6 million (≥approximately USD 4800). It was then recategorized into two groups: KRW < 1.5 million (approximately USD < 1200) and KRW ≥ 1.5 million (approximately USD ≥ 1200). Education level was divided into two groups: those who had not completed high school and those who had. Participants who were either never smokers or had quit smoking were classified as non-smokers, whereas those who smoked either occasionally or habitually were classified as current smokers. Alcohol consumption was categorized as current drinking or non-drinking. Height (cm) and weight (kg) were measured by trained staff using standardized tools and techniques (such as wearing minimal clothing, positioning the instrument on a flat surface, and reading the value when it was fixed) [33]. Body mass index (BMI) was computed by dividing body weight in kilograms by the square of the height in meters. A questionnaire was used to determine the physical activity level of the participants. The time spent exercising per day was assigned a weight based on the intensity of the exercise (light, moderate, and vigorous), which enabled the calculation of metabolic equivalents of task-hours per week [34].

### 2.3. Dietary Information and PI

During the baseline survey conducted between 2001 and 2002 and the second follow-up survey conducted in 2005–2006, the participants’ food and nutrient intakes were assessed by trained staff using a Semi-Quantitative Food Frequency Questionnaire (SQFFQ). SQFFQs are validated and reproducible tools that assess the frequency and amount of food intake over the last year [35]. The food list for the SQFFQ was based on data from the 1998 Korea National Health and Nutrition Examination Survey [36]. The KARE baseline survey included 103 food items, while the second follow-up survey included 106 food items. The intake frequency of each food item was recorded on a 9-stage scale, ranging from rarely eaten to three times a day, and the intake amount was recorded on a 3-stage scale, ranging from small to large. Nutrient intake, including energy and sodium, was calculated as the average daily intake level of the baseline and second follow-up data to minimize the possibility of misclassification of dietary information [36].

For this analysis, a phytochemical database was developed using the National Standard Food Composition Table ver 10.0 from the Rural Development Administration [37] and the Computer-Aided Nutritional Analysis Program (5.0) from the Korean Nutrition Society [38]. The PI was estimated based on average intake levels of the baseline and a 2nd follow-up SQFFQ, and the PI was computed by dividing the energy obtained from consuming phytochemical-rich foods by the total daily energy intake, and then expressing this ratio as a percentage [26,27]. The PI included identical food items to those included in previous studies [27], such as whole grains, vegetables, fruits, legumes, nuts and seeds, soybeans and soybean products, and seaweed. Additionally, green tea and black coffee, which are frequently consumed by this population, were included in the PI calculation, as recent studies have demonstrated that these beverages contain significant amounts of phytochemicals [39,40]. Pickles, potatoes, sweet potatoes, and salted vegetables such as kimchi were excluded from the PI calculation due to their inconsistent representation in the dietary database and variability in phytochemical content [41].

Other dietary habits related to gastritis/gastric ulcer (such as preference for spicy or sweet foods) were collected through a survey consisting of five responses, wherein the participants rated their degree of liking from “strongly dislike” to “strongly like”. These dietary habits were assessed to determine their potential role as etiological factors or in exacerbating symptoms of existing disease. The proportion of total energy from carbohydrate, protein, and fat intake were calculated as follows: ([each nutrient intake (g) × energy of each nutrient per 1 g (kcal)] ÷ [total energy intake (kcal)]) × 100.

### 2.4. Definition of Gastritis/Gastric Ulcer Diagnosis

Gastritis and gastric ulcer are both inflammatory conditions of the gastric mucosa and share similar etiological factors such as *Helicobacter pylori* infection, use of nonsteroidal anti-inflammatory drugs (NSAIDs), smoking, and alcohol consumption. Both conditions can present with overlapping clinical symptoms and require similar diagnostic and therapeutic approaches.

The data for these two conditions were collected through a single question and therefore were combined for analysis. Gastritis/gastric ulcer was diagnosed based on a survey questionnaire administered by trained staff [33], and was defined as meeting one or more of the following criteria: physician diagnosis, prior treatment experience, active treatment, previous use of medication, current medication regimen, or hospitalization.

### 2.5. Statistical Analysis

The follow-up period was defined as the time between (1) the date of baseline survey and the date of diagnosis of gastritis/gastric ulcer, or (2) the date of baseline survey and the last follow-up of participants who did not develop gastritis/gastric ulcer (i.e., last known survival). The incidence rate was calculated by dividing the number of participants who developed gastritis/gastric ulcer during the follow-up period by the sum of the follow-up periods (in years) for all participants. The distribution of participants’ characteristics based on the PI quartile was analyzed using the chi-square test for categorical variables with frequency and percentage and general linear regression for continuous variables with mean and standard error. The association between PI quartiles and gastritis/gastric ulcer risk was evaluated using Cox proportional hazards regression with hazard ratio (HR) and 95% confidence interval (CI). The median of each quartile was used as a continuous variable in the regression analysis to calculate *p* for trend. Potential confounding factors were investigated based on a preliminary analysis and literature review, [11,13,14,17], and three statistical models were built as follows: Model 1 (unadjusted) Model 2 (adjusted for sex and age), and Model 3 (adjusted for variables including sex, age, household income, education level, smoking status, alcohol consumption, physical activity, BMI, total energy intake, sodium intake, and preference for spicy and sweet foods). Effect modifiers that may affect the association between PI and gastritis/gastric ulcer risk were tested using a multiplicative term in the regression model, and no significant effect modifiers were observed. Non-parametric restricted cubic spline regression analysis was conducted to test for linear and nonlinear associations between PI and gastritis/gastric ulcer after adjusting for all covariates included in Model 3. All analyses were conducted using SAS version 9.4 (statistical analysis system; SAS Institute Inc., Cary, NC, USA) [42] with a statistical significance level of α = 0.05.

## 3. Results

The median follow-up period was 9.50 years, during which the incidence of gastritis/gastric ulcer was 13.94 per 1000 person-years.

### 3.1. General Characteristics of the Participants

Table 1 presents the general characteristics of the study participants at baseline, stratified by PI quartile. The median PI values for quartiles 1, 2, 3, and 4 were 6.27, 10.80, 15.46, and 21.29, respectively. Participants with higher PI values were generally older (*p* < 0.001) and female (*p* < 0.001), had lower levels of education (*p* = 0.001) and household income (*p* = 0.002), were non-smokers (*p* < 0.001), non-drinkers (*p* < 0.001), and engaged in moderate physical activity (*p* < 0.001). In addition, high PI values were associated with higher levels of BMI (*p* = 0.006).

### 3.2. Dietary Habits of the Participants

Table 2 presents the dietary habits of the study participants according to the quartiles of their PI values. Higher PI values were associated with a higher proportion of total energy from protein intake (*p* < 0.001) and higher sodium intake (*p* < 0.001). In contrast, lower PI values were associated with higher mean scores for preference of spicy food (*p* < 0.001).

### 3.3. Association between Quartiles of PI and Gastritis/Gastric Ulcer Risk

Table 3 presents the HR for gastritis/gastric ulcer according to the quartiles of PI. In the unadjusted Model 1, there was no significant association between PI and the risk of gastritis/gastric ulcer (HR: 0.89, 95% CI: 0.72–1.11, *p* for trend = 0.14). However, after adjusting for sex and age in Model 2, PI was significantly and inversely associated with the risk of gastritis/gastric ulcer (HR: 0.79, 95% CI: 0.63–0.99, *p* for trend = 0.01). In the fully adjusted Model 3, the association was stronger, indicating a 22% lower risk of gastritis/gastric ulcer for those in the fourth quartile compared to those in the first quartile of PI (HR: 0.78, 95% CI: 0.61–0.98), and this association was linear, indicating a reduced risk with increasing PI quartile (*p* for trend = 0.01).

### 3.4. Dose–Response Relationship between PI and Gastritis/Gastric Ulcer Risk

**Figure 1:**
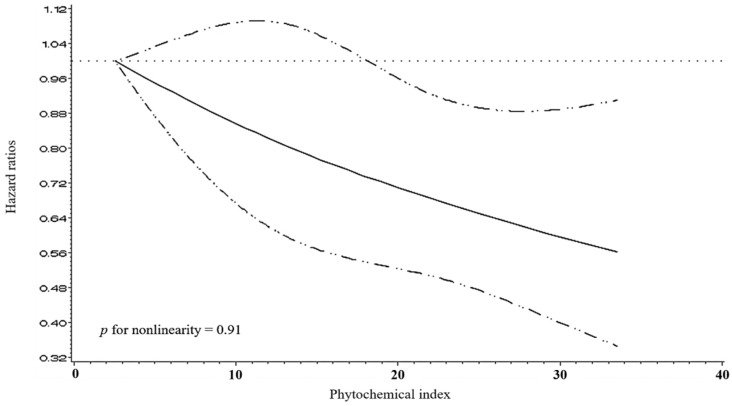
Multivariable-adjusted hazard ratios (95% confidence intervals) for the non-parametric relationship between the PI and gastritis/gastric ulcer. The model was adjusted for sex (men and women), age (continuous), household income (lower or mid-low and mid-high or higher), education level (lower than high school education and high school educated or higher), smoking status (current smokers and non-smokers), alcohol consumption (current drinkers and non-drinkers), physical activity (low, mid, and high), body mass index (continuous), total energy intake (continuous), sodium intake (continuous), preference for spicy food (categorical), and preference for sweet food (categorical).

The spline curve in Figure 1 shows a dose–response relationship between PI and gastritis/gastric ulcer, after adjusting for covariates, including sex, age, household income, education level, smoking status, alcohol consumption, physical activity, BMI, total energy intake, sodium intake, preference for spicy food, and preference for sweet food. The results indicate a strong linear relationship, demonstrating a reduction in risk with an increase in PI (*p* for nonlinearity = 0.91).

## 4. Discussion

The findings indicate that a higher PI is significantly associated with a lower risk of gastritis/gastric ulcer in Korean adults; further, a dose–response relationship was observed, suggesting that the risk of developing gastritis/gastric ulcer decreased as PI increased.

Phytochemicals are plant compounds with bioactive non-nutritive properties [20]. They are found in foods such as whole grains, fruits, and vegetables and are associated with reduced risk of chronic diseases [20,27]. Several cross-sectional studies have demonstrated that a high intake of phytochemical-rich foods is associated with lower inflammation levels and a lower prevalence of metabolic syndrome, obesity/abdominal obesity, and hypertension in Koreans [27,28,29,30]. Moreover, the consumption of plant foods, such as fruits and vegetables, has been linked to a reduced risk of precancerous lesions in the stomach, such as atrophic gastritis or dysplasia [43,44]. For instance, a study in Japan showed that individuals who consumed light-colored vegetables ≥4 times a week had a lower prevalence of chronic atrophic gastritis than those who consumed light-colored vegetables ≤3 times a week [43]. Similarly, a study in Venezuela found that those who consumed fruits <7 times a week had a higher prevalence of chronic atrophic gastritis and dysplasia compared to those who consumed fruits ≥14 times a week [44]. These findings highlight the importance of consuming a diet rich in phytochemicals and plant-based foods to promote good health and prevent chronic diseases.

The potential beneficial effects of diets rich in phytochemicals in regard to gastric ulcers and gastritis may be explained by the antioxidant and anti-inflammatory properties of phytochemicals, which reduce reactive oxygen species (ROS) and regulate the biosynthetic pathways of prostaglandins [22,45]. Gastritis and gastric ulcer are types of inflammatory diseases [1,46], and inflammation can be induced by the production of ROS related to the activation of the nuclear transcription factor kappa B, which regulates the expression of cytokines [47,48,49]. Furthermore, the gastrointestinal tract is known to be a key source of ROS, and oxidative stress is associated with several gastrointestinal diseases [45,46]. Phytochemicals with antioxidant and anti-inflammatory properties may have positive effects on gastric diseases [22,23,24,25]. For example, carotenoids are known for their singlet oxygen-scavenging abilities [19,20], while flavonoids reduce ROS and inhibit free radical production through the chelation of metal ions, such as free Fe and Cu [22,45]. Flavonoids also exhibit antiulcer effects by increasing bicarbonate secretion and inhibiting pepsin levels and activity; their gastric cytoprotective activity can be exerted through the regulation of the prostaglandin biosynthetic pathway [22]. Additionally, chalcones with one or more isoprenyloxyl groups can exert a cytoprotective effect by stimulating the synthesis of mucous substances in the gastric mucosa and increasing mucosal blood flow, and more [22,23]. Polyphenols are known to have the fuvanction of interfering with oxidative stress signal transduction and inhibit pro-inflammatory signaling [21,24,25]. They also possess antioxidant and immunomodulatory properties and inhibit the formation of N-nitroso compounds [24,50]. Green tea polyphenols have been reported to inhibit the release of tumor necrosis factor-α [51], while quercetin, a type of polyphenol, can activate the nuclear erythroid 2-related factor 2-antioxidant response element pathway, which is involved in neuroprotection against oxidative damage and apoptosis [24,25,52]. Sulforaphane, which is found in cruciferous vegetables such as broccoli, stimulates various antioxidant enzymes, including glutathione S-transferase and heme oxygenase-1 [53]. These properties are speculated to make phytochemicals effective in protecting against gastric diseases.

Although this study yielded compelling results, it has some limitations. First, although the study accounted for potential confounding factors by conducting a comprehensive literature review and preliminary analysis, there might still be residual confounding variables that were not known or measured, or certain types of variables (e.g., categorical variables), owing to the limitations of the study design. For instance, factors such as *Helicobacter pylori* infection status, use of NSAIDs, family history of gastritis/gastric ulcer, and the severity of gastritis/gastric ulcer are potential confounding variables in the association between PI and gastric diseases; however, these factors were not measured or were unavailable for analysis. Second, this study is that the PI was based on the energy content of the consumed foods. Therefore, low- or non-energy foods rich in phytochemicals may not have been fully accounted for, leading to an underestimation of phytochemical intake levels. Furthermore, the study could not examine specific types of phytochemicals due to the lack of a comprehensive phytochemical database, indicating a need for further research. Third, although SQFFQs, a validated tool for dietary assessment, was used to construct the PI, SQFFQs are known to rely heavily on the participant’s memory, which can introduce recall issues. These recall issues may be greater when compared to other dietary assessment methods such as 24 h dietary recalls or food records, which are generally less dependent on long-term memory and provide more immediate and detailed dietary information. To mitigate these potential biases in our study, the SQFFQ was administered according to a standardized protocol by trained investigators. This approach was designed to ensure consistency and reduce variability in the data collection process, enhancing the reliability of our findings. Fourth, this study had a small sample size, which precluded subgroup analysis. Due to the limited number of participants, it was not feasible to conduct detailed analyses for different subgroups (e.g., by age, gender, or specific health conditions). Future studies with larger sample sizes are recommended to explore these aspects in more detail. Fifth, this study relied on self-reported data for exposure, outcomes, and most covariates. While self-reported data are valuable, they are susceptible to recall bias and inaccuracies, which could potentially affect the validity of the diagnosis and the overall findings of the study. Specifically, diagnosing gastritis and gastric ulcers based on self-reported data (including physician diagnosis, prior treatment experience, active treatment, previous use of medication, current medication regimen, or hospitalization) may not be as accurate as endoscopic confirmation. This limitation could lead to misclassification, as endoscopy is necessary to improve diagnostic accuracy. Lastly, the study was conducted using data from the Korean population, and the PI was calculated based on phytochemical-rich foods frequently consumed by Koreans. This limits the generalizability of the findings to other populations with different dietary patterns.

## 5. Conclusions

In conclusion, the present study found a significant inverse association between PI and the risk of gastritis/gastric ulcer in Korean adults, suggesting that consuming a diet rich in phytochemicals may help reduce the risk of these inflammatory diseases. These findings provide valuable insights for future dietary guidelines and emphasize the need for further large-scale epidemiological studies to confirm this association and explore the underlying mechanisms.

## Figures and Tables

**Table 1:** Baseline characteristics of the KoGES ^a^ study population according to the quartiles of dietary PI ^b^ (*n* = 7377).

	Quartiles of Dietary PI				*p* ^†^
	1 (Low)	2	3	4 (High)	
No. of participants	1844	1844	1845	1844	
PI, median (range)	6.27 (0.13–8.58)	10.80 (8.59–13.07)	15.46 (13.08–17.91)	21.29 (17.92–64.22)	
Age (years), Mean ± SE ^c^	51.26 ± 0.21	51.55 ± 0.20	52.25 ± 0.21	54.46 ± 0.21	<0.001
Sex, N (%)					<0.001
Men	1254 (68.00)	1023 (55.48)	781 (42.33)	524 (28.42)	
Women	590 (32.00)	821 (44.52)	1064 (57.67)	1320 (71.58)	
Education level, N (%)					0.001
Lower than high school education	1003 (54.81)	1002 (54.55)	1015 (55.25)	1104 (60.39)	
High school educated or higher	827 (45.19)	835 (45.45)	822 (44.75)	724 (39.61)	
Household income ^d^, N (%)					0.002
Lower or mid-low	928 (51.10)	888 (48.66)	890 (49.09)	989 (54.34)	
Mid-high or higher	888 (48.90)	937 (51.34)	923 (50.91)	831 (45.66)	
Smoking status, N (%)					<0.001
Non-smokers	1117 (60.97)	1282 (70.17)	1448 (79.30)	1562 (85.40)	
Current smokers	715 (39.03)	545 (29.83)	378 (20.70)	267 (14.60)	
Alcohol consumption, N (%)					<0.001
Non-drinkers	750 (40.76)	884 (48.20)	1027 (55.94)	1189 (64.80)	
Current drinkers	1090 (59.24)	950 (51.80)	809 (44.06)	646 (35.20)	
Body mass index (kg/m^2^), Mean ± SE	24.56 ± 0.07	24.65 ± 0.07	24.76 ± 0.07	24.83 ± 0.08	0.006
Physical activity ^e^, N (%)					<0.001
Low	625 (34.23)	590 (32.33)	625 (34.13)	602 (32.80)	
Mid	541 (29.63)	593 (32.49)	617 (33.70)	673 (36.68)	
High	660 (36.14)	642 (35.18)	589 (32.17)	560 (30.52)	

^a^ KoGES, Korean Genome and Epidemiology Study; ^b^ PI, phytochemical index; ^c^ SE, standard error. ^†^ *p* values were calculated using the chi-square test for categorical variables and *p* for trends across quartiles of the dietary PI were calculated using linear regression models for continuous variables. ^d^ Household income was divided into Korean republic won (KRW) < 1.5 million (lower or mid-low) and KRW ≥ 1.5 million (mid-high or higher). Conversion factor: 1 KRW = 0.0008 US dollars. ^e^ Physical activity level was grouped into tertiles based on the level of metabolic equivalent task-hours per week.

**Table 2:** Dietary habits of the study population according to the quartiles of dietary PI (*n* = 7377).

	Quartiles of Dietary PI				*p* for Trend
	1 (Low)	2	3	4 (High)	
No. of participants	1844	1844	1845	1844	
Total energy intake (kcal/day)	1813 ± 11.38	1933 ± 12.58	1944 ± 12.28	1840 ± 12.12	0.26
Carbohydrate (% of total energy)	72.18 ± 0.15	71.26 ± 0.14	71.16 ± 0.14	71.77 ± 0.14	0.07
Protein (% of total energy)	12.45 ± 0.05	13.18 ± 0.05	13.56 ± 0.05	13.93 ± 0.05	<0.001
Fat (% of total energy)	13.70 ± 0.12	14.32 ± 0.11	14.29 ± 0.11	13.74 ± 0.10	0.89
Sodium intake (mg/day)	2876.13 ± 29.97	3060.70 ± 30.17	3100.31 ± 32.89	3122.62 ± 33.38	<0.001
Preference for spicy food ^a^	3.30 ± 0.02	3.14 ± 0.02	3.14 ± 0.02	2.99 ± 0.02	<0.001
Preference for sweet food ^a^	2.95 ± 0.02	3.01 ± 0.02	2.98 ± 0.02	2.95 ± 0.02	0.64

PI, phytochemical index. Values are mean ± standard error. ^a^ The preference for spicy and sweet food was assessed using a five-point Likert scale, with each response option scored and presented as the mean ± standard error: strongly dislike (1), dislike (2), neutral (3), like (4), and strongly like (5).

**Table 3:** Hazard ratios (95% confidence intervals) for gastritis/gastric ulcer according to the dietary PI quartile (*n* = 7377).

	Quartiles of Dietary PI				*p* for Trend
	1 (Low)	2	3	4 (High)	
No. of participants	1844	1844	1845	1844	
No. of cases (%)	183 (9.92)	211 (11.44)	180 (9.76)	155 (8.41)	
Model 1 ^a^	Reference	1.06 (0.87–1.29)	0.90 (0.74–1.11)	0.89 (0.72–1.11)	0.14
Model 2 ^b^	Reference	1.02 (0.83–1.24)	0.83 (0.67–1.02)	0.79 (0.63–0.99)	0.01
Model 3 ^c^	Reference	1.01 (0.82–1.24)	0.82 (0.66–1.02)	0.78 (0.61–0.98)	0.01

PI, phytochemical index. ^a^ Model 1, unadjusted. ^b^ Model 2, adjusted for sex (men and women), and age (continuous). ^c^ Model 3, Model 2 additionally adjusted for household income (lower or mid-low and mid-high or higher), education level (lower than high school education and high school educated or higher), smoking status (current smokers and non-smokers), alcohol consumption (current drinkers and non-drinkers), physical activity (low, mid, and high), body mass index (continuous), total energy intake (continuous), sodium intake (continuous), preference for spicy food (categorical), and preference for sweet food (categorical).

## Data Availability

The KoGES data used in this study are available upon request and subject to approval. Interested researchers may apply for access via the National Institute of Health Korea website (https://www.nih.go.kr), where applications will be reviewed and approved accordingly.
